# Transcriptomic and proteomic analyses of *Mangifera indica* in response to *Xanthomonas critis* pv. *mangiferaeindicae*

**DOI:** 10.3389/fmicb.2023.1220101

**Published:** 2023-07-04

**Authors:** Feng Liu, Xin Sun, Lulu Wang, Kaibing Zhou, Quansheng Yao, Ru-lin Zhan

**Affiliations:** ^1^Key Laboratory of Hainan Province for Postharvest Physiology and Technology of Tropical Horticultural Products, Key Laboratory of Tropical Fruit Biology, Ministry of Agriculture, South Subtropical Crops Research Institute, Chinese Academy of Tropical Agricultural Sciences, Zhanjiang, Guangdong, China; ^2^College of Horticulture, Hainan University, Haikou, China

**Keywords:** mango bacterial leaf spot, proteomics, transcriptomics, plant hormone signaling, cellular redox homeostasis

## Abstract

Mango is an important tropical fruit with the reputation of “Tropical Fruit King.” It is widely cultivated in tropical and subtropical regions. Mango bacterial leaf spot, which is caused by *Xanthomonas critis* pv. *mangiferaeindicae* (*Xcm*), poses a great threat to the development of mango planting industry. In this study, we used RNA sequencing and data-independent acquisition techniques to compare the transcriptome and proteome of the highly resistant cultivar “Renong No.1” (RN) and the highly susceptible cultivar “Keitt” (KT) in response to *Xcm* infection at different stages (0, 2, and 6 days). A total of 14,397 differentially expressed genes (DEGs) were identified in the transcriptome of the two varieties, and 4,400 and 8,926 genes were differentially expressed in RN and KT, respectively. Among them, 217 DEGs were related to plant hormone signaling pathway, and 202 were involved in the maintenance of cellular redox homeostasis. A total of 3,438 differentially expressed proteins (DEPs) were identified in the proteome of the two varieties. Exactly 1,542 and 1,700 DEPs were detected in RN and KT, respectively. In addition, 39 DEPs were related to plant hormone signaling pathway, whereas 68 were involved in the maintenance of cellular redox homeostasis. Through cross-validation of the two omics, 1,470 genes were found to be expressed in both groups, and a large number of glutathione metabolism-related genes, such as *HSP26-A*, *G6PD4*, and *GPX2*, were up-regulated in both omics. Peroxisome-related genes, such as *LACS6*, *LACS9*, *PED1*, *GLO4*, and *HACL*, were up-regulated or down-regulated in both omics. *ABCB11*, *SAPK2*, *MYC2*, *TAG7*, *PYL1*, and other genes related to indole-3-acetic acid and abscisic acid signal transduction and plant-pathogen interaction were up-regulated or down-regulated in both omics. We also used weighted gene co-expression network analysis to combine physiological and biochemical data (superoxide dismutase and catalase activity changes) with transcriptome and proteome data and finally identified three hub genes/proteins (*SAG113*, *SRK2A*, and *ABCB1*) that play an important role in plant hormone signal transduction. This work was the first study of gene/protein changes in resistant and susceptible mango varieties, and its results improved our understanding of the molecular mechanism of mango resistance to *Xcm*.

## Introduction

Mango (*Mangifera indica* L.) is a kind of evergreen tree, originated in Malaysia, India. It has a long history of planting in China and is an important agricultural industry in tropical regions. Its fruit is not only rich in nutrients such as vitamin A, vitamin C and amino acids, but also its branches, leaves and peels contain a large number of bioactive substances, such as polyphenols, terpenes, carotene and phytosterols, which have certain edible value and medical value (Lebaka et al., 2021). Studies have shown that these active substances in mango have anti-inflammatory, immunomodulatory, antibacterial, anti-diabetic, anti-obesity and anti-cancer effects in medicine (Mirza et al., 2021).

Mango bacterial leaf spot (MBLS), which is caused by *Xanthomonas critis* pv. *mangiferaeindicae* (*Xcm*), can cause serious damage to fruit health, which results in reduced or zero mango yield. At present, disease-resistance breeding is the most economical and effective control method for disease resistance (Zandalinas et al., 2021). Therefore, studying the changes in gene and protein expression in mango during *Xcm* infection can not only lead to complete understanding of the molecular mechanism of mango resistance to MBLS but also provide valuable genetic resources for the breeding of disease-resistant mango varieties.

Plant hormones, such as ethylene (ETH), jasmonic acid (JA), salicylic acid, auxin, indole-3-acetic acid (IAA), abscisic acid (ABA), and gibberellin (GA), are key regulators of plant immunity (Li et al., 2019). They interact in complex networks to respond to pathogen invasion and thus exhibit resistance to pathogens (Denancé et al., 2013). Anderson et al. (2004) observed that the transcription level of *AtMYC2*, a positive regulator of ABA signal transduction in *Arabidopsis thaliana*, was induced in the early stage of soil-borne pathogenic fungus *Fusarium oxysporum* infection by reverse transcription-quantitative polymerase chain reaction (PCR). Further overexpression of *AtMYC2* showed that the levels of ETH and JA were significantly lower than those in the control group, which indicates the antagonistic effect of ABA on JA and ETH. Their interaction regulated the expression of *Arabidopsis* defense and stress genes in response to biological stress. Li et al. (2013) used Illumina technology to analyze the transcriptome changes of roots of Cavendish banana varieties infected with *Fusarium oxysporum* f. sp. *Cubense* (*Foc*). The two genes encoding ETH biosynthesis enzyme aminocyclopropanecarboxylate oxidase and several ETH-responsive transcription factors were one of the strongly induced genes of *Foc*, which indicates that ETH synthesis and signaling pathways were activated in response to Foc infection. Djami-Tchatchou et al. (2022) conducted a global transcriptomic analysis of tomato strain DC3000 (*Pto*DC3000) and observed that IAA inhibited the expression of genes involved in the type III secretion system and exercise; thus, IAA is a signal molecule for gene expression in *Pto*DC3000.

Under pathogen attack, reactive oxygen species (ROS) will accumulate in plants, and excessive accumulation will cause serious damage to plant proteins, DNA, and other cellular components, thus promoting the invasion of pathogens (Sies, 2018). At this point, the enzymatic systems, including catalase (*CAT*), superoxide dismutase (*SOD*), glutathione peroxidase (*GPX*), and glutathione S-transferase (*GST*), and non-enzymatic system, such as ascorbic acid, glutathione (GSH), mannitol, and flavonoids, play important roles in plants (Bela et al., 2015; Meitha et al., 2020). Xue et al. (2020) discovered that after phytoplasma caused red date witch broom disease, the genes involved in GSH cycle and thioredoxin synthesis in jujube leaves were up-regulated at the transcriptional and metabolic levels. The activities of *GST* and *GPX* in disease-resistant varieties were higher than those in susceptible varieties, which indicates that the antioxidant defense system plays an important role in plant pathogen invasion. Akbar et al. (2020) reported differences in the transcription levels of ROS-related genes between the disease-resistant sugarcane variety (B-48) infected with *Sugarcane mosaic virus* and the susceptible sugarcane variety (Badila). Compared with Badila, the expression of *GST* was significantly reduced, whereas those of transcription factors, such as *WRKY*, *AP2*, and *bHLH*, were significantly increased in B-48. Therefore, the genes involved in the ROS detoxification pathway can be used as key indicators for pathogen attack in plants.

Next-generation RNA sequencing (RNA-Seq) and data-independent acquisition (DIA) are currently the most advanced high-throughput technologies, and they can perform global analysis of gene and protein expressions in a large number of biological samples. Joint analysis of transcriptome and proteome is widely used to address plant responses to various biotic stresses. Cucumber fusarium wilt caused by *Fusarium oxysporum f*. sp. *cucumerinum* (*FOC*) is one of the most important diseases in cucumber cultivation. In the exploration of the molecular mechanism of cucumber response to *FOC* infection, combined transcriptome and proteome analyses of cucumber leaves inoculated with *FOC* at 2 and 4 days showed that *FOC* infection activated plant hormone signals and transcription factors and inhibited wax biosynthesis and photosynthesis. The accumulation of redox proteins also plays a key role in cucumber resistance to *FOC* (Xie et al., 2022). Kiwifruit is an important tropical fruit in China. Kiwifruit bacterial canker caused by *Pseudomonas syringae* pv. *Actinidiae* (*Psa*) is an important disease in the kiwifruit seed industry. The transcriptome and proteome analyses of the resistant variety “Jinkui” and the susceptible variety “Hongtao” showed that the pathways of “phytohormone signal transduction” and “phenylpropanol biosynthesis” were activated at the protein and transcriptional levels after *Psa* infection. The transient expression of *AcMYB16* gene in “Jinkui” induced *Psa* infection (Wang et al., 2021). However, reports on the response of mango to *Xcm* are limited.

At present, the research on MBLS mainly focuses on the comprehensive treatment of MBLS and the isolation and identification of MBLS pathogens (Gagnevin and Pruvost, 2001; Sanahuja et al., 2016). The research on the molecular mechanism of mango resistance to MBLS is still in its infancy. This work is the first to study the changes in gene and protein expressions in mango during *Xcm* infection. Our findings will provide new ideas for MBLS resistance and valuable genetic resources for the breeding of MBLS-resistant mango.

## Methods

### Preparation of bacterial solution

Single colonies of activated *Xcm* cultured for 48 h were picked into LB and incubated at 200 rmp at 28°C for 2 days before inoculation. The concentration of pathogen was about 1 × 10^9^ CFU/mL determined by plate colony counting method.

### Treatment of plant material

The resistant and susceptible mango varieties “Renong No.1” (RN) and “Keitt” (KT) were used as plant materials. *Xcm* was identified by pathogenicity determination, morphology, and 16S ribosomal RNA (16S) from the susceptible leaves of KT mango in the mango germplasm resource nursery of the South Subtropical Crops Institute of the Chinese Academy of Tropical Agricultural Sciences. We selected healthy fruits with the same size and maturity, soaked them in 1% sodium hypochlorite for 2 min for disinfection, washed them thrice with sterile water, and then placed them in an alcohol-disinfected plastic box to dry naturally. Plum blossom needles were used for acupuncture inoculation, and 60 μL mixed bacterial solution was added at each inoculation point. The inoculated fruits were placed in a fresh keeping box at 28°C and 100% humidity to be sampled, and the same treatment with LB liquid medium was used for the control. Each fruit was inoculated in three places, seven points were inoculated in each place, and three fruits were inoculated as triplicate. On days 0, 2, and 6 of inoculation with *Xcm*, the mango epidermis with a thickness of 1–2 mm on the surface of the inoculation point was used as the experimental sample and stored at −80°C for use.

### SOD and CAT analyses

For the determination of superoxide dismutase (SOD) and catalase (CAT) activities, based on the ratio of experimental sample weight (g):volume (mL) = 1:9, phosphate buffer solution 9 times the volume of the sample was added (0.1 mol/L, pH 7.0–7.4). Then, the sample was homogenized in an ice water bath and centrifuged at 12,000 rpm for 15 min at 4°C, and the supernatant was collected for measurement. An ultraviolet spectrophotometer or a Tecan Spark microplate reader was used to measure the absorbance value of the reaction solution, and the result was inputted into the formula to calculate the SOD and CAT activities. The analyses at each time point were repeated thrice.

### RNA extraction, library construction, and sequencing

Total RNA was extracted using Trizol reagent kit (Invitrogen, Carlsbad, CA, USA), in accordance with the manufacturer’s protocol (Poyraz et al., 2010). RNA quality was assessed on an Agilent 2100 Bioanalyzer (Agilent Technologies, Palo Alto, CA, USA) and checked using RNase-free agarose gel electrophoresis. After the total RNA was extracted, eukaryotic mRNA was enriched by Oligo(dT) beads. Then, the enriched mRNA was broken into short fragments using fragmentation buffer and reverse transcribed into cDNA using NEBNext Ultra RNA Library Prep Kit for Illumina sequencing (NEB #7530, New England Biolabs, Ipswich, MA, USA) (Salmela and Rivals, 2014). The purified double-stranded cDNA fragments were end repaired, added with A base, and ligated to Illumina sequencing adapters. The ligation reaction was purified with AMPure XP Beads (1.0X). The ligated fragments were subjected to size selection by agarose gel electrophoresis and PCR amplification. The resulting cDNA library was sequenced using Illumina Novaseq6000 by Gene *Denovo* Biotechnology Co. (Guangzhou, China).

### Transcriptome data analysis

The raw readings produced by transcriptome sequencing were quality controlled by fastp (version 0.18.0) (Chen et al., 2018), and the comparison tool Bowtie (version 2.2.8) (Langmead and Salzberg, 2012) was used to remove low-quality reads. Then, the clean reads were compared with the mango genome of each sample by HISAT (version 2.2.4) (Kim et al., 2015). No more than three base mismatches were observed. To analyze the gene expression in mango, mango variety “Hong Xiang Ya” was used as the reference genome,^1^ the total number of valid reads obtained from all samples was 1,456,435,960, and the number of reads that could be mapped to the mango genome was 1,325,116,148. After counting the reads for each gene, the set of genes expressed in each time period was counted for each cultivar, and differences between cultivars were analyzed by Venn diagram. At the same time, the sample cluster diagram was used to cluster the samples in different time periods to ensure the reliability of the subsequent analysis results. Finally we used the fragments per kilobase of transcript per million mapped reads (FPKM) method for normalization (Li and Dewey, 2011). Low-expression genes were filtered (<5 FPKM), and RNA differential expression analysis was performed between two different groups by DESeq2 (Love et al., 2014) (and by edgR between two samples) (Ashburner et al., 2000; Robinson et al., 2010). The genes with false discovery rate (FDR) below 0.05 and absolute fold change (FC) ≥ 2 were considered differentially expressed genes (DEGs).

### Protein sample preparation

Sample preparation involved protein denaturation, reduction, alkylation, tryptic digestion, and peptide cleanup. Commercially available iST Sample Preparation kit (PreOmics GmbH, Planegg, Germany) was used following the protocols provided. Briefly, after the samples were ground with liquid nitrogen, 50 μL lysis buffer was added and heated at 95°C for 10 min at 1,000 rpm with agitation. After cooling the sample to room temperature, trypsin digestion buffer was added, and the sample was incubated at 37°C for 2 h at 500 rpm with shaking. The digestion process was stopped with a stop buffer. Sample clean-up and desalting were carried out in the iST cartridge using the recommended wash buffers. Peptides were eluted with elution buffer (2 × 100 μl) and then lyophilized by SpeedVac.

### DIA protein detection

Before mass spectrometry detection, Biognosys quality control reagent from the iRT Kit was added to each sample, and calibration was performed based on the retention time of the polypeptide in chromatography. QuiC (Biognosys) software was used to control the original mass spectrometry data to investigate the similarity in the quality control indicators between each sample. If the index results were similar, the detection repeatability was good. Then, Pulsar software was used to build a database of the data obtained from the date-dependent acquisition (DDA) mode, and the date-independent acquisition (DIA) data were analyzed based on the DDA reference database to identify proteins. When at least one sample detected a protein, the qualitative results of the protein and quantitative results in all samples were outputted.

Qualitative analysis of proteins was conducted to detect proteins in the sample and identify their types. To ensure the reliability of results, we checked whether protein qualitative analysis results meet the following identification criteria: precursor threshold of 1.0% FDR and protein threshold of 1.0% FDR at the peptide and protein levels, respectively. The average peak area of the first three MS1 peptides with the FDR of less than 1.0% was screened for protein quantification.

After counting the reads for each protein, the set of genes expressed in each time period was counted for each cultivar, and differences between cultivars were analyzed by Venn diagram. Finally, according to the results of protein quantification, the proteins with significant changes in abundance between the comparison groups were screened. Statistical test FDR value and fold change log2FC were used to screen proteins with significant differences. The default threshold was FDR < 0.05, |log2(fc)| > 0.58. This part can visualize the results of difference analysis in the form of chart interaction.

### Functional analysis

Gene Ontology (GO) enrichment analysis provided all GO terms that were significantly enriched in DEGs/differentially expressed proteins (DEPs) compared with the genome background, whereas the DEGs/DEPs that corresponded to biological functions were filtered (Young et al., 2010). First, all DEGs/DEPs were mapped to the GO terms in the GO database,^2^ and gene and protein numbers were calculated for every term (Chen et al., 2017). Significantly enriched GO terms in DEGs/DEPs compared with the genome background were defined by hypergeometric test. Kyoto Encyclopedia of Genes and Genomes (KEGG)^3^ is a major public pathway-related database (Kanehisa and Goto, 1999; Fang et al., 2021). Pathway enrichment analysis identified significantly enriched metabolic pathways or signal transduction pathways in DEGs/DEPs compared with the whole genome or proteome background. The formula was the same as that in GO analysis (Kanehisa and Goto, 1999). The calculated *p*-value was subjected to FDR correction, with FDR ≤ 0.05 as a threshold (Anders and Huber, 2010).

### Network construction

To identify genes or proteins related to CAT, and SOD changes, we performed a weighted gene co-expression network analysis (WGCNA) on the genes and proteins. Co-expression networks were constructed using WGCNA (v1.47) package in R (Langfelder and Horvath, 2008). After filtering genes and proteins (<1 reads per kilobase per million mapped reads), gene/protein expression values were imported into WGCNA to construct co-expression modules using the automatic network construction function BlockwiseModules with default settings, except that the power was 13, and minimum module size was 50. Genes/proteins were clustered into 20 correlated modules (Botía et al., 2017).

### Module and gene selection

To detect biologically significant modules, we used module eigengenes to calculate the correlation coefficient with samples or sample traits. Correlation analysis was performed using a module eigengene with data for specific traits or phenotypes (Niemira et al., 2019). Pearson correlation between each gene and trait data under the module were also calculated for the most relevant module (positive and negative correlations) corresponding to each phenotype data.

### Quantitative RT-PCR analysis

Premier 6 was used to design gene qRT-PCR primers based on the CDS sequence of the differential genes (Supplementary Table 1). The reverse transcribed C-DNA of total RNA (the same sample as transcriptome sequencing) of mango fruit inoculated with the pathogen of mango bacterial keratitis at 0, 2, and 6 days was used as the template. The mango actin gene was used as the reference gene, and the dye kit method was used to verify the results. The entire RNA reverse transcription step was performed according to the reverse transcription kit instructions (Taylor et al., 2019). Expression was calculated using the 2^–ΔΔCt^ method (Livak and Schmittgen, 2001).

## Results

### Symptoms after LB and *Xcm* treatments

**FIGURE 1:**
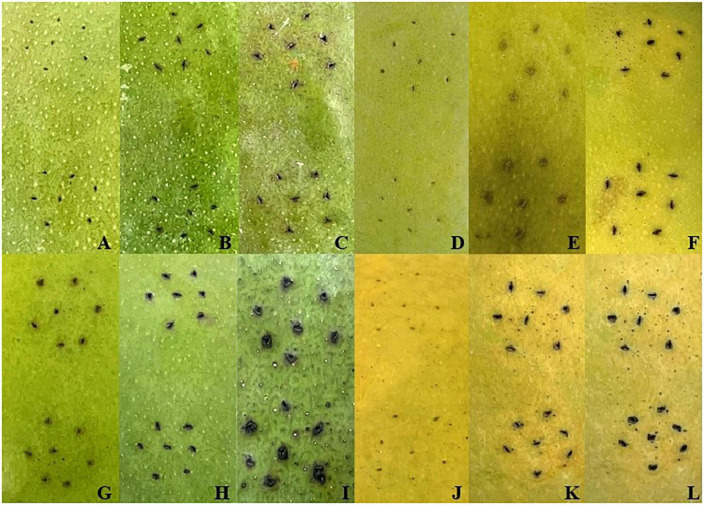
Symptoms after LB and *Xcm* treatments. Panels **(A–C)** and **(D–F)** were the symptoms of “Keitt” and “Renong No.1” after 0 days, 2 days, and 6 days of LB liquid medium treatment, respectively. Panels **(G–I)** and **(J–L)** were the symptoms of “Keitt” and “Renong No.1” after 0 days, 2 days, and 6 days of treatment with bacterial solution containing *Xcm*, respectively.

After inoculation with *Xcm* and LB liquid medium by needling method, the phenotypic changes of the two varieties were observed at 0, 2 and 6 days (Figure 1). It was found that after inoculation with LB liquid medium, the color of the inoculation point of “Keitt” and “Renong No.1” gradually turned brown over time, but the changes at the inoculation point of the two varieties were not obvious. During the whole experimental period of *Xcm* inoculation, the changes of symptoms of the two varieties were consistent with their disease resistance. The specific manifestations were as follows: on day 2 after inoculation, black spots began to appear at the inoculation sites of the two varieties, and the symptoms were similar at this time. On the sixth day after inoculation, the symptoms of the two varieties at the indirect seeding sites were obviously different. The black spots of “Renong 1” were deepened and slightly spread. “Kate” was more widespread, showing the typical ‘crisscross volcanic’ pattern of bacterial corner spot in mango orchards. The results suggest that 0, 2, and 6 days after *Xcm* inoculation may be key sampling time points to explore the mechanisms of resistance to bacterial keratosis in mangoes with different resistance.

### Overview of mango fruit transcriptome

To study the changes in gene expressions in fruits of KT and RN after inoculation with *Xcm*, we obtained the pericarp tissues of two resistant and susceptible germplasms at 0, 2, and 6 days after inoculation with pathogens. Then, we used TRIzol reagent to extract the total RNA of each sample and sequenced them by Illumina HiSeq 2000 platform. Initially, transcriptome sequencing generated about 59,160,000 original reads and about 59,040,000 clean reads for all samples. Then, the clean reads were aligned with the mango genome sequence, which resulted in 90.15–92.13% clean reads with no more than three base mismatches. To analyze mango gene expressions, we calculated the number of clean reads aligned with mango gene sequences (36,065 sequences) and normalized them using the FPKM method. After filtering the low-expression genes (<5 FPKM), we identified 28,704 genes (about 79.59% of all mango genes) in all samples.

After counting the readings, to exclude the influence of genetic background differences between KT and RN on subsequent analyses, we analyzed the expression of KT and RN overall genes under LB treatment. A Venn diagram (Supplementary Figure 1) showed that 7,057 genes were expressed, and no genes were detected that were expressed specifically at a single time point. This ruled out the possibility of misinterpretation of gene expression data generated during the natural growth of mango.

### DEGs in resistant and susceptible mango fruits

**FIGURE 2:**
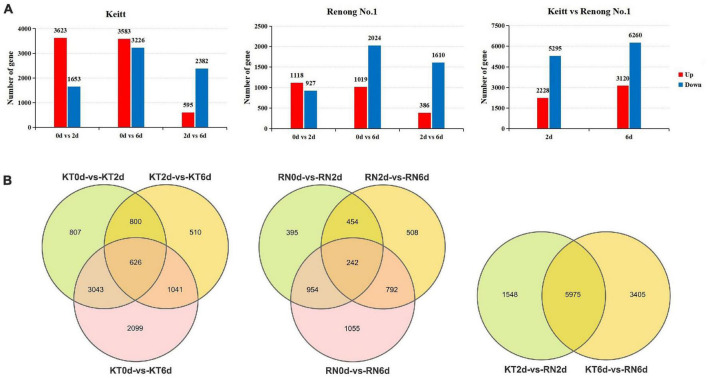
DEGs of RN and KT. Panel **(A)** is the expression summary of DEGs in RN and KT, red represents upregulation and blue represents downregulation; Panel **(B)** is the distribution of DEGs in KT and RN, the left is DEGs in KT, the middle is the DEGs in RN, and the right is DEGs in RN at different stages after inoculation (relative to KT).

To analyze mango fruit gene expression, we used Deseq2 software to calculate *P*- and FDR values and the default FDR < 0.05; | log2FC| > 1 indicated differential genes. We identified 14,397 DEGs in the KT and RN. A total of 8,926 DEGs were identified in KT. Compared with KT0d, 5,276 (3,623 up-regulated and 1,653 down-regulated) and 6,809 DEGs (3,583 up-regulated and 3,226 down-regulated) were identified in KT2d and KT6d, respectively. Compared with KT2d, we identified 2,977 DEGs in KT6d (595 up-regulated and 2,382 down-regulated). We identified 4,400 DEGs in RN. Compared with RN0d, we identified 2,045 (1,118 up-regulated and 927 down-regulated) and 3,043 DEGs (1,019 up-regulated and 2,044 down-regulated) in RN2d and RN6d, respectively. Compared with RN2d, we identified 1,996 DEGs (386 up-regulated and 1,610 down-regulated) in RN6d. In the further comparison of RN2d with KT2d and RN6d with KT6d, we identified 7,523 (2,228 up-regulated and 5,295 down-regulated) and 9,380 DEGs (3,120 up-regulated and 6,260 down-regulated) in the RNs, respectively (Figure 2A). Venn diagram (Figure 2B) showed that 626 DEGs were continuously differentially expressed during the whole infection period of KTs, and 242 DEGs were continuously differentially expressed during the whole experimental period of RNs. Furthermore, 5,975 DEGs were shared between KTs and RNs. A total of 3,650 out of 8,926 DEGs identified in KTs (40.89%) and 2,355 out of 4,400 DEGs identified in RNs (53.52%) were specifically expressed on day 6, which indicated that the transcriptome of mango changed significantly on day 6 after *Xcm* infection.

**FIGURE 3:**
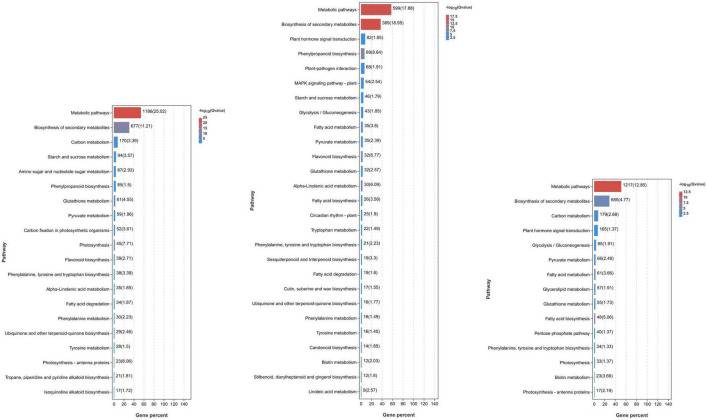
Significant KEGG pathways of DEGs identified in RN and KT. Different pathways and their *p*-values of the number of genes contained and the degree of enrichment significance were plotted, and only the pathway that met the threshold was selected for plotting. Each column represents a pathway, and the color of the column represents the enrichment significance of that Pathway. The numbers outside the brackets next to the columns represent the number of genes belonging to the pathway in the module. The values in parentheses next to the columns represent the degree of enrichment and are −log10(*p*-value) and correspond to the color of the columns. In the significance bars, the order “from large to small” was selected according to the number of genes enriched in the pathway.

To understand the possible pathways and functions of these DEGs in mango response to *Xcm*, we performed GO and KEGG enrichment analyses on the DEGs (Figure 3; Supplementary Tables 2, 3). GO enrichment analysis showed that a large number of DEGs in KTs and RNs were annotated to metabolic process (GO: 0008152), cellular process (GO: 0009987), catalytic activity (GO: 0003824), biological regulation (GO: 0065007), response to stimulus (GO: 0050896), membrane (GO: 0016020), and cell part (GO: 0044464). These significantly enriched GO terms were associated with the symptoms of MBLS, including “cross-shaped volcanic lesions” and black spots. KEGG enrichment analysis revealed that a large number of DEGs of RNs and KTs are involved in GSH metabolism, phenylalanine metabolism, peroxisome, and other important pathways. However, several differences were observed in the pathways involved in certain DEGs enriched by KTs and RNs. Specific pathways were only found in RN enrichment results, and these pathways included plant mitogen-activated protein kinase signaling pathway, plant-pathogen interaction, plant hormone signal transduction, and cutin, suberin, and wax biosynthesis. Thus, the DEGs of RNs and KTs are involved in different pathways in response to *Xcm* infection.

### Genes in the plant hormone signaling pathway

A total of 217 DEGs were found to be related to plant hormone signal transduction pathway (Supplementary Table 4). These DEGs included 63 IAA-related genes, such as auxin-responsive protein (*IAA26*, *LAX2*, *ARF9*, and *AUX22D*) and IAA amido synthetase (*GH3.1*, *GH3.6*, and *GH3.10*), of which 22 were down-regulated in RN and KT. Twelve genes were associated with ABA; two of them (*ABF2* and *PYL8*) decreased, and three (*DPBF2*, *PYL1*, and *PYL3*) increased in RN and KT. A total of 6 and 14 ETH-related genes (*EIL3*, *EIL1*, *EIN3*, and *ETR1*) decreased in KT and RN, respectively, and the remaining 8 genes showed different expression trends in various mango varieties. A total of 12 genes were related to GA, 4 (*GAIPB*, *GID1B*, *GID2*, and *GAI*) were down-regulated in RN and KT, and the remaining 7 genes showed different expression patterns in various mango species. In addition, 10 and 17 TF genes (1 *TGA*, 2 *PIF*, 5 *MYC*, and 1 *HBP-1b*) were down-regulated in RN and KT, respectively. A total of 7 were down-regulated (4 serine/threonine-protein kinase, 1 *MKK*, and 2 *BAK*), and 3 kinases (*MKK*, serine/threonine-protein kinase, and *AHK*) were up-regulated. The up-regulation and down-regulation of these genes indicated that hormone signaling was induced by *Xcm* infection in mango tissues, and the levels of plant hormones may play an important role in this process.

### Genes involved in the maintenance of cellular redox homeostasis

Among the 14,397 DEGs, 202 were found to be involved in the redox process or play a regulatory role in cellular redox homeostasis (Supplementary Table 5). A total of 74 genes were annotated to peroxisomes, and 32 genes were up-regulated in RN but down-regulated in KT. CAT isozyme (*CAT1*), Nudix hydrolase family (*NUDT15*, *NUDT19*), and other enzymes were annotated as fatty acyl-CoA reductase, phytanoyl-CoA dioxygenase, and long chain acyl-CoA synthetase. A total of 81 DEGs were involved in GSH metabolism, including those of L-ascorbate peroxidase (*APX3*, *APXS* and *APXT*), glucose-6-phosphate 1-dehydrogenase (*G6PD2*, *G6PD4*, and *G6PDH*), and 20 of them were continuously up-regulated in RN; their expression levels were significantly higher than those in KT. After *Xcm* infection, 18 redox proteins (3 CAT isozymes, 10 ferredoxin (Fd), and 5 SOD) were induced, among which *CAT1*, *FD3*, and *Os07g0147900* were up-regulated in RN and down-regulated in KT. The expression levels of 11 photosystem I (PSI) reaction center subunits and 8 PSII proteins generally increased in resistant and susceptible varieties, but the changes in KT were more significant.

### Overview of mango fruit proteome

Proteomics technology is widely used in the study of protein differential expression and various post-translational modifications (Haverland et al., 2014; Sidoli et al., 2015). In this study, we used DIA (a new holographic quantitative technique based on electrostatic field orbitrap) to investigate the protein expression changes in KT and RN mango during *Xcm* infection (0, 2, and 6 days). To ensure the reliability of the results, we checked whether the protein qualitative analysis findings met the identification criteria, namely, precursor threshold of 1.0% FDR and protein threshold of 1.0% FDR, at the peptide and protein levels, respectively. Finally, 12,260 peptides and 12,877 proteins were identified from the two mango varieties.

As with the transcriptome analysis, after counting, we analyzed KT and RN total protein expression under LB treatment. The Venn diagram (Supplementary Figure 2) shows that 11,329 proteins were co-expressed across all tested time points, and no more than 35 proteins were specifically expressed at each tested time point. For reliability of subsequent analysis, 11,329 co-expressed proteins were selected for subsequent analysis.

### DEPs in resistant and susceptible mango fruits

**FIGURE 4:**
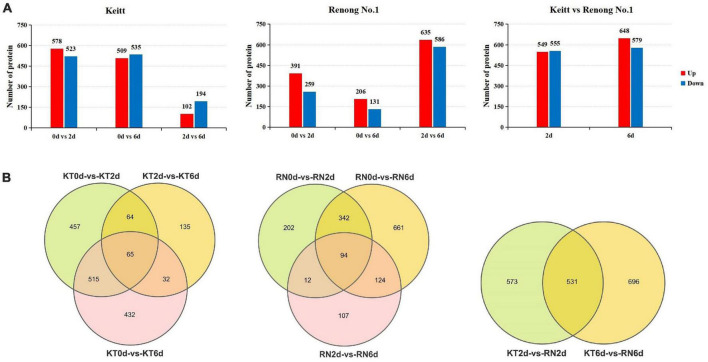
DEPs of RN and KT. Panel **(A)** is the expression summary of DEPs in RN and KT, red represents upregulation and blue represents downregulation; Panel **(B)** is the distribution of DEPs in KT and RN, the left is DEPs in KT, the middle is the DEPs in RN, and the right is DEPs in RN at different stages after inoculation (relative to KT).

We aimed to understand the differences in protein expression levels in response to *Xcm* in different mango varieties. According to the screening threshold of DEPs, the absolute value of the FC was greater than 1.5 times (| log2(1.5)| ≈0.58 corrected *P*-value (Q value) < 0.05). The proteins with significant differences between groups (KT0d vs. KT2d, KT0d vs. KT6d, KT2d vs. KT6d, RN0d vs. RN2d, RN0d vs. RN6d, RN2d vs. RN6d, KT2d vs. RN2d, and KT6d vs. RN6d) were screened. A total of 1,700 DEPs were identified in KT. Compared with KT0d, 1,101 (578 up-regulated and 523 down-regulated) and 1,044 (509 up-regulated and 535 down-regulated) DEPs were identified in KT2d and KT6d, respectively. Compared with KT2d, 296 DEPs were identified in KT6d (102 up-regulated and 194 down-regulated). A total of 1,542 DEPs were identified in RNs. Compared with RN0d, 650 (391 up-regulated and 259 down-regulated) and 337 DEPs (206 up-regulated and 131 down-regulated) were identified in RN2d and RN6d, respectively. Compared with RN2d, 1,221 DEPs (635 up-regulated and 586 down-regulated) were identified in RN6d (Figure 4A). The Venn diagram (Figure 4B) showed that 65 and 94 DEPs were significantly differentially expressed after KTs and RNs were infected with *Xcm*, respectively. A total of 1,800 DEPs were detected between KTs and RNs. Similar to the transcriptome results, the expression of DEPs induced on the 6th day of *Xcm* infection was higher than that of all DEPs. A total of 1,243 DEPs (50.92%) were observed in KT6d and 1,340 (60.69%) in RN6d. Thus, the 6th day of *Xcm* infection not only caused great changes in the transcriptome of mango but also the expression of its proteins.

**FIGURE 5:**
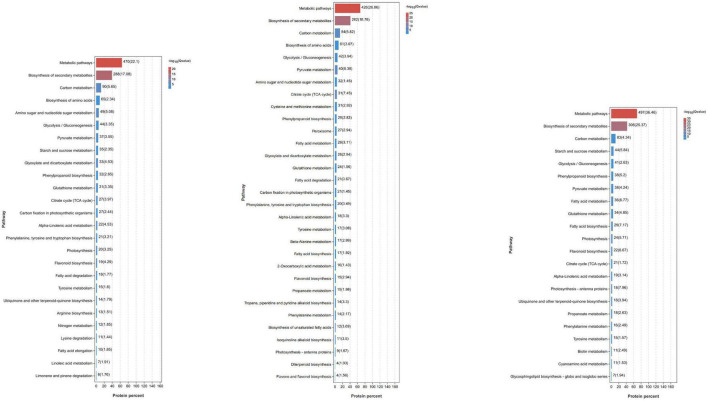
Significant KEGG pathways of DEPs identified in RN and KT. Different pathways and their *p*-values of the number of proteins contained and the degree of enrichment significance were plotted, and only the pathway that met the threshold was selected for plotting. Each column represents a pathway, and the color of the column represents the enrichment significance of that pathway. The numbers outside the brackets next to the columns represent the number of proteins belonging to the pathway in the module. The values in parentheses next to the columns represent the degree of enrichment and are −log10(*p*-value) and correspond to the color of the columns. In the significance bar, the order “from large to small” was selected according to the number of enriched proteins in the pathway.

To understand the function of DEPs in RNs and KTs and the pathways involved in regulation, we also conducted GO and KEGG enrichment analyses on DEPs (Figure 5; Supplementary Tables 6, 7). GO enrichment results showed a large number of organism metabolic process proteins in both cultivars (GO: 0044710), including oxoacid metabolic process (GO: 0043436), cellular homeostasis (GO: 0019725), regulation of hormone levels (GO: 0010817), and other biological processes, such as oxidoreductase activity (GO: 0016491), catalytic activity (GO: 0003824), antioxidant activity (GO: 0016209), and other molecular functions that neutralize cell-cell junction (GO: 0005911); cell periphery (GO: 0071944), cytoplasmic part (GO: 0044444), and other cellular components. We speculate that *Xcm* may act mainly on the membrane of mango cells or accelerate the process of infection by secreting special substances or degrading normal mango cell structures to bind them to cells. KEGG enrichment analysis showed that a large number of proteins are involved in metabolic pathways, biotin metabolism, carbon metabolism, and other pathways. DEPs in RNs were also significantly enriched in important pathways, such as peroxisome and GSH metabolism. We suggest that mango infection may trigger a series of physiological and chemical reactions, such as the synthesis of plant hormones and lignin, antioxidant production, and changes in ROS.

### Proteins in the plant hormone signaling pathway

Similar to the transcriptome analysis, we identified 39 DEPs associated with plant hormone signaling pathway (Supplementary Table 8). Among these DEPs, several were identified together with their transcriptomes, two were associated with IAA (*IAA26* and *LAX2* were up-regulated in RN and down-regulated in KT), seven were associated with ABA, and six (*PYL1*, *PYL2*, 2 *PYL9*, and 2 *ABF2*) were generally down-regulated in KT and RN. One DEP (*PYL8*) was up-regulated in RN but down-regulated in KT. Several transcription factors (*TGA7*, *TGA21*, and *MYC2*) were down-regulated in RN and KT, and the proteome-specific protein *TGAL1* was identified (down-regulated in RN and up-regulated in KT). In addition, we observed that eight classes of kinases (*BSK7*, *SAPK3*, *SRK2A*, *SRK2E*, *BSK2*, *SAPK2*, *ASK7*, and *BSK1*) (*BSK1* and *ASK7* were up-regulated in RN and down-regulated in KT, and the others were down-regulated in KT and RN). The pathogenesis-related protein *PRB1* was up-regulated in KT and RN but was more evident in RN. The findings indicate that the regulatory pathways involving several transcription factors and kinases and IAA and ABA signaling pathways are the keys to mango response to *Xcm*.

### Proteins involved in the maintenance of cellular redox homeostasis

A total of 68 DEPs were identified to be related to the maintenance of cellular redox homeostasis (Supplementary Table 9). These DEPs included 5 redox proteins (3 *FD3* and 2 *CAT1* were up-regulated in RN but down-regulated in KT; *FSD2* was down-regulated in KT and RN), of which 38 are involved in the production of peroxisomes, including *PED1*, *PMP22*, *ACX3*, *ACX4*, and *ACX2*, and were up-regulated in KT and RN, with a stronger response observed in RN. A total of 49 genes are related to GSH metabolism, and 29 genes, such as GST (*HSP26-A*, *GSTU7* and *PARC*), 6-phosphogluconate dehydrogenase (*PGD3*), and phospholipid hydroperoxide GSH (*GPX1* and *GPX2*), were up-regulated in RN and KT. Nine PSI- and three PSII-related proteins, such as *PSAA*, *PSAH2*, and *PSB27-1*, were continuously down-regulated in RN but were significantly increased in KT on day 6 after *Xcm* stress. Proteins involved in the regulation of antioxidant enzymes and peroxisome biosynthesis were also identified in the transcriptome, and the expression patterns of PS-related proteins differed between KT and RN. These results indicate that proteins from these three pathways may play important roles in the resistance of mango to *Xcm* invasion.

### Cross-validation of transcriptomics and proteomics

**FIGURE 6:**
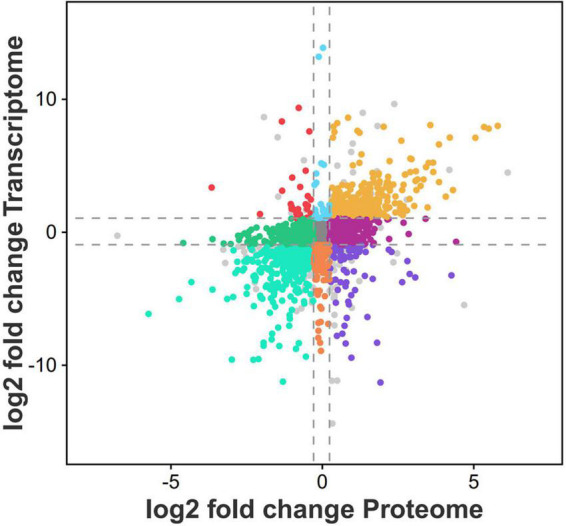
Nine quadrant map of common differential genes between transcriptome and proteome. The abscission is the log2 value of protein fold change, and the ordinate is the log2 value of transcriptome fold change. The dashed line represents the log2 value of the difference multiple specified in the difference analysis. In the figure, each colored point represents the collection of a class of genes, in which the colored points represent genes that meet the differential analysis threshold range (that is, meet the screening conditions for differential mRNA and differential protein), and the gray points represent genes that do not meet the analysis threshold.

To screen the gene set with the same or opposite expression trend in the two groups, based on the results of transcriptome and proteome difference analyses, we selected the common genes in the two omics for nine-quadrant analysis (Figure 6). The results showed that 1,470 genes were detected in the differential analysis of the two omics (Supplementary Table 10), and the expression patterns of 663 DEGs and DEPs were consistent (306 up-regulated and 357 down-regulated). The expression patterns of 95 DEGs were opposite those of DEPs (24 were up-regulated at the transcriptional level and down-regulated at the protein level; 71 were up-regulated at the protein level and down-regulated at the transcriptional level). A total of 580 genes were differentially expressed only at the protein level (271 up-regulated and 309 down-regulated). In addition, 132 genes were only differentially expressed at the transcriptional level (34 up-regulated and 98 down-regulated). Notably, a large number of DEGs/DEPs, including GST (*HSP26-A* and *PARC*), glucose-6-phosphate 1-dehydrogenase (*G6PD2* and *G6PD4*) and GPX (*GPX2*), were found to be associated with GSH metabolism. Some DEGs/DEPs were also found to be associated with peroxisomes, including co-upregulated (*LACS6*, *LACS9*, and *PED1*) and co-downregulated (*GLO4* and *HACL*) genes, which are involved in the regulation of cellular redox homeostasis and protect cells from stress-induced oxidative damage. Four ABC transporter families, including *ABCB11*, *ABCB26*, and *ABCB28*, were up-regulated in both omics. *MYC2* and *TGA7* transcription factors related to plant IAA and ABA signal transduction were down-regulated in both omics. *PYL1* and *PYL9* were down-regulated, and *SAPK2* was up-regulated in both omics; these genes are related to plant ABA signaling and plant-pathogen interaction.

### Differential accumulation of SOD and CAT in two mango cultivars after *Xcm* inoculation

Transcriptome and proteome analyses showed that SOD and CAT constantly run through them. Thus, these antioxidant enzymes are significant for mango response to *Xcm*. Therefore, the activities of SOD and CAT in diseased mango fruits were determined at different time points (0, 2, and 6 days) (Table 1).

**TABLE 1:** Data statistics for CAT and SOD.

Name of sample	Activity of SOD (U/g FW)	Activity of CAT (U/g FW)
KT0-CK-1	116.7192	146.1181
KT0-CK-2	101.1579	147.3061
KT0-CK-3	108.9385	146.7121
RN0-CK-1	68.7029	101.7278
RN0-CK-2	61.1496	116.3664
RN0-CK-3	75.1006	109.0471
KT2-CK-1	51.3421	146.4533
KT2-CK-2	43.0272	150.7463
KT2-CK-3	47.1847	148.5999
RN2-CK-1	64.8143	103.0125
RN2-CK-2	69.9055	106.6258
RN2-CK-3	73.3733	104.8196
KT6-CK-1	127.6464	137.9829
KT6-CK-2	95.9465	122.1177
KT6-CK-3	111.7965	130.0503
RN6-CK-1	123.1138	126.6759
RN6-CK-2	92.8053	121.7515
RN6-CK-3	107.9596	116.8271
KT2-1	60.9331	221.6349
KT2-2	77.7148	220.1284
KT2-3	56.0119	229.9195
RN2-1	96.6945	151.8826
RN2-2	83.0136	153.2427
RN2-3	89.8540	152.5627
KT6-1	318.2606	207.4966
KT6-2	407.8865	209.8200
KT6-3	363.0736	208.6583
RN6-1	591.5970	314.2697
RN6-2	467.4140	307.9936
RN6-3	529.5055	311.1317

The results showed that SOD and CAT activities increased in both cultivars after *Xcm* inoculation at different times. In addition, significant differences (*P*-value < 0.0001) were observed in the changes of the same index among different varieties (Supplementary Figure 3). The results indicated that the two varieties may have different response mechanisms to *Xcm* stress.

### Co-expressed genes/proteins by WGCNA

**FIGURE 7:**
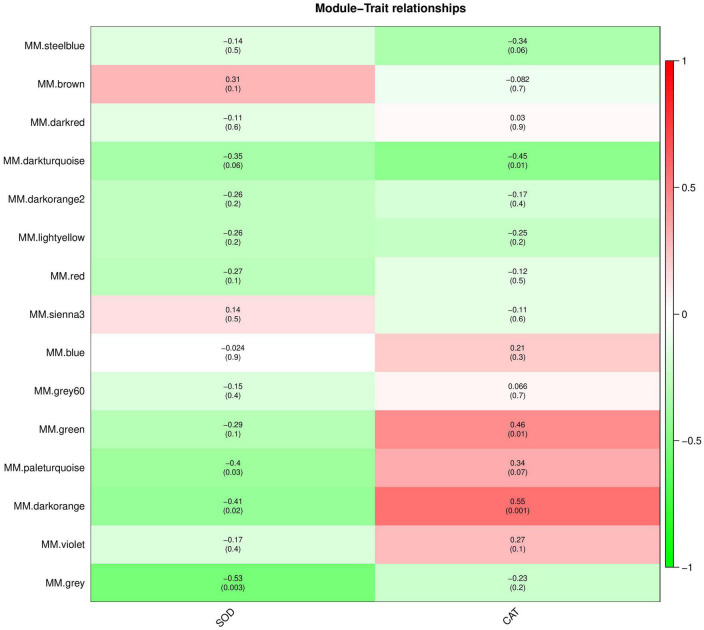
Module-trait relationships of co-expressed genes. The abscissa is the trait, the ordinate is the module, and the module eigenvalues and trait data are plotted using Pearson correlation coefficients. In the figure, red represents positive correlation, green represents negative correlation, and darker colors indicate stronger correlation.

**FIGURE 8:**
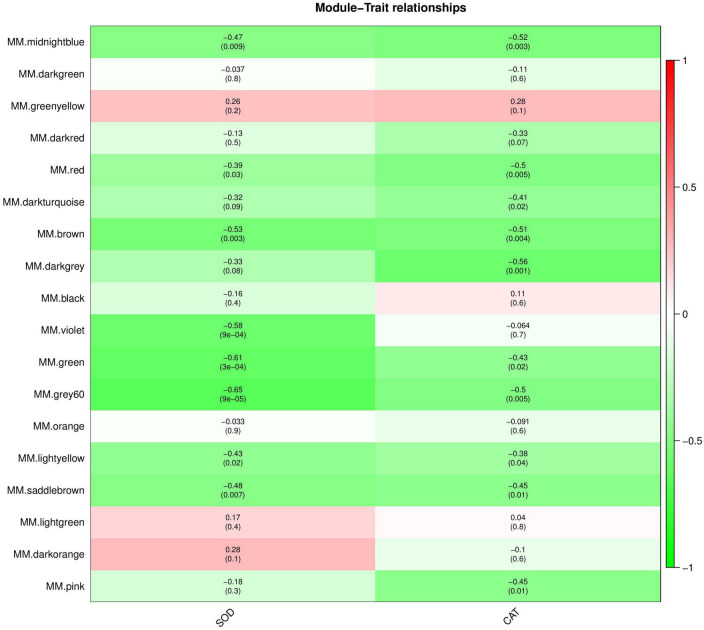
Module-trait relationships of co-expressed proteins. The abscissa is the trait, the ordinate is the module, and the module eigenvalues and trait data are plotted using Pearson correlation coefficients. In the figure, red represents positive correlation, green represents negative correlation, and darker colors indicate stronger correlation.

The interaction between plants and pathogens usually leads to the rapid accumulation of ROS in plants (Fichman and Mittler, 2021; Mittler et al., 2022). Several antioxidant enzymes play a key role in detoxification of ROS produced by plant stress response (Czarnocka and Karpiński, 2018). Therefore, we used WGCNA to screen the genes and proteins with the strongest correlation with SOD and CAT activity changes to further understand the co-expression relationship between KT and RN genes/proteins related to antioxidant enzymes. We selected the module with the highest Pearson r between the module and physiological and biochemical data as the key module. Removing the outlier grey module, in the gene co-expression network analysis, we observed that the brown and dark orange gene modules were most positively/negatively correlated with the SOD activity change pattern (brown: cor = 0.31, *P*-value = 0.1; dark orange: cor = −0.41; *P*-value = 0.02), containing 2,062 and 259 genes, respectively. The dark-orange and dark-turquoise gene modules were most positively/negatively correlated with CAT activity change patterns (dark orange: cor = 0.55, *P*-value = 0.001; dark-turquoise: cor = −0.45, *P*-value = 0.01). The dark-turquoise gene module contained 176 genes (Figure 7). In the protein co-expression network analysis, the dark-orange and grey60 modules exhibited the most positive/negative correlation with the change pattern of SOD activity (dark orange: cor = 0.28, *P*-value = 0.1; grey60 module: cor = −0.65, *P*-value = 9e-05), containing 217 and 180 proteins, respectively. The green-yellow and dark-gray modules had the most positive/negative correlation with CAT activity change pattern (green-yellow: cor = 0.28, *P*-value = 0.1; darkgray: cor = −0.56, *P*-value = 0.001), containing 261 and 131 proteins, respectively (Figure 8). KEGG pathway analysis showed that all three co-expressed gene modules were involved in “Glutathione metabolism,” which plays an important role in plant antioxidant and integrated detoxification functions (Bela et al., 2015). We also found 17 genes involved in “Peroxisome,” which is an important component of the plant antioxidant system, in the brown module (Yun and Kim, 2018). Next, we analyzed the co-expressed protein modules. The dark-orange module was annotated to “Metabolic pathways” and “Carbon metabolism,” grey60 to “Spliceosome” and “RNA degradation,” and green-yellow to “Flavonoid biosynthesis.” The dark-gray module was annotated to “Phenylpropanoid biosynthesis,” “Biotin metabolism,” and “Diterpenoid biosynthesis.” These findings indicate that the gene/protein modules are involved in different pathways to respond to *Xcm* invasion and may play an important role in maintaining cellular oxidative balance and biological regulation through these pathways.

Comparison of WGCNA results identified 58 co-expressed genes/proteins at the mRNA and protein levels. A total of 58 genes were compared with the DEGs identified in this study (Table 2). Exactly 20 and 19 DEGs were detected in KT and RN, respectively, of which 8 were dysregulated in RN (Table 3). Compared with DEPs, 31 proteins were differentially expressed in two mango cultivars; 13 and 11 of these proteins were differentially expressed in KT and RN, respectively, and 5 were dysregulated in RN (Table 4). These results indicate that differential genes/proteins may interact with non-differential ones. These proteins are not only related to mango antioxidant and certain biosynthesis but also may be related to mango disease resistance.

**TABLE 2:** A total of 58 co-expressed genes and proteins were identified by WGCNA results.

	Gene expression level							Protein expression level					
**Id**	**Symbol**	**RN0d**	**RN2d**	**RN6d**	**KT0d**	**KT2d**	**KT6d**	**RN0d**	**RN2d**	**RN6d**	**KT0d**	**KT2d**	**KT6d**
mango000296	CYP98A2	9.077	45.163	7.223	28.54	135.043	29.203	120449.669	336072.583	400800.042	254158.284	1019853.219	1696598.542
mango000839	SAG113	932.533	670.88	624.033	254.743	335.347	309.333	631877.458	455194.052	434339.406	683416	167780.609	124673.814
mango003892	CYP75A1	3.343	75.497	13.91	35.847	129.567	34.317	236681.745	1493866.958	2342342.417	339446.219	5058423.667	4947420.667
mango004150	THFS	49.22	60	38.217	32.26	67.733	37.697	11576418.67	9818077.333	11823384	9814331.667	8291622	9367840
mango005596	CYP86A22	46.457	53.637	64.88	2.973	9.297	16.103	1515010.354	816778.292	253823.823	850163.25	169501.807	259646.812
mango005775	ELF5	29.387	26.813	26.037	0.1	0.257	0.077	608604.5	437408.49	448061.917	652486.052	603007.802	461195.115
mango006296	4CL2	15.937	81.573	25.067	39.437	185.467	45.13	267768.224	1657512.75	2795836.583	225517.089	6310093.667	7322742.833
mango006430	GC5	28.737	35.537	45.947	16.92	19.077	14.26	551177.49	346006.497	168726.192	210471.427	239766.174	272378.44
mango006938	At3g47520	102.743	106.617	161.383	51.213	36.82	49.63	11182567.33	11850842	13976115.67	8119996.667	6680809.833	7291565.667
mango007487	UAH	9.41	12.19	7.76	5.117	10.513	5.913	3059991.667	2540401.833	2867465.25	2088687.208	1974811.583	2077584.917
mango008372	GLIP5	39.963	49.117	35.507	20.78	20.93	17.393	639855.354	623948.417	819532.771	334714.906	301998.797	376429.344
mango008492	CKL1	25.397	26.27	31.577	0.073	0.247	0.033	425939.167	230623.167	228819.406	379393.182	145321.557	149100.297
mango008818	BRIZ1	0.763	0.573	0.557	29.807	22.913	18.11	240898.339	298488.969	178607.548	225951.594	329491.573	254470.734
mango009044	–	68.547	109.253	32.673	43.683	132.607	40.69	2286412.917	2726363.583	2348346.917	1447527.583	3331601.917	4125099.167
mango011025	MC410	29.327	16.913	17.647	0.047	0.09	0	130390.026	88184.142	66171.405	99272.096	60717.603	54723.872
mango012295	RCOM_ 1506700	10.373	14.467	21.237	4.343	7.463	8.597	802806.979	708689.208	841368.208	675084.917	411557.135	555602.979
mango012298	MVD2	60.73	79.223	100.39	30.483	30.51	27.407	4598980.5	4295668.75	5038793	3759557.167	3429621.583	3287508.333
mango012341	SPBC776.07	4.817	5.737	3.673	9.85	9.967	9.53	1180885.667	976157.25	1187463.333	892162.625	776807.417	950840.375
mango012352	RTM2	0.507	0.387	3.96	0.253	0.073	0	435499.844	464847.885	331642.167	416058.344	276312.526	242228.646
mango012477	tal	393.79	559.74	637.897	233.253	289.607	195.803	11053258	10469292	13088137	8039736	7254753.667	7475896.5
mango013678	KAS1	52.033	76.32	97.343	39.407	26.873	28.02	4089943.333	2669365.333	3076852	3255481.833	2750220.333	3036436.417
mango013911	–	99.383	116.91	143.463	41.58	56.157	66.46	3339037.083	3102724	2848404.833	3595689.167	1778566.875	2227503.875
mango015130	SRK2A	13.223	8.907	10.103	39.007	35.01	35.897	364706.802	245055.76	173405.807	365675.781	201554.737	184828.219
mango017964	RTNLB8	17.583	23.377	14.03	9.523	25.83	13.333	892079.771	1053576.51	265746.654	1506117.625	1298243.458	600708.497
mango018070	SUMO2	3.64	4.283	3.76	1.68	0.97	2.07	605803.062	425804.167	416404.781	501482.458	307870.026	337847.292
mango018604	GF14D	164.24	222.787	174.74	125.323	252.17	166.763	32110846	26634130.67	29684760.67	23849617.33	23407323.33	29048290
mango018984	PAE8	20.477	114.597	16.65	6.75	90.6	9.203	14259275.67	9416066	9666316.333	9980805	10416728	8661339.167
mango020228	AFB2	58.567	62.18	89.82	37.887	38.09	32.177	544816.146	423586.354	326912.156	523882.51	422021.208	267861.823
mango020536	DBR	14.047	3.567	1.4	2.497	4.22	5.127	1327042.792	963911.958	489440.458	1111487.875	531932.365	427321.708
mango021031	HEXO2	15.157	32.947	11.707	26.427	34.927	16.99	151324.245	439039.583	416883.708	372989.229	879524.708	819515.25
mango021699	PLT5	19.467	54.62	37.61	30.247	99.233	26.93	667121.625	738229.729	662353.208	269335.333	1002994.146	1141336.271
mango022055	ABCB20	22.193	17.357	14.127	31.92	29.63	28.647	352198.042	398142	246857.323	426489.229	410300.302	325395.583
mango022508	ADCK1	48.847	15.487	10.243	36.453	24.97	23.143	147165.857	192104.661	181896.562	156913.062	298447.896	278558.891
mango023351	HCS1	45.403	41.58	51.943	22.843	21.87	23.69	520459.594	426767.615	297574.5	490239.083	337919.406	299679.25
mango023529	XK2	11.023	14.383	24.583	8.207	15.717	13.29	4484826.25	3297040.083	5104332.333	3658099.167	3117518.25	3054485.167
mango023835	SCP2	65.787	92.243	79.777	19.347	51.837	41.58	1972901.833	1787336.958	1749565.875	2421409.417	1098470.312	1508311.292
mango024212	At5g42250	1.323	4.267	3.45	2.543	5.167	3.46	160343.128	154011.594	295552.198	197642.305	342618.052	528385.219
mango024861	TFT7	63.79	67.6	75.36	10.187	27.02	29.803	730657.021	629114.354	746246.938	404433.219	380753.344	407370.042
mango026117	PGL3	10.34	8.013	13.213	4.267	5.69	4.417	547663.844	484599.74	445614.302	547793.781	242155.807	113928.124
mango026233	POR1	171.973	175.867	175.513	71.66	87.68	90.32	5275425.5	5293905.667	6443040.667	3263134.917	3106708.333	3300088.25
mango027123	At2g47970	78.183	63.787	38.19	89.54	96.82	83.147	984970.667	688723.688	593986.25	568024.156	611011.042	705327.167
mango027688	EO	804.393	1005.29	2212.13	40.2	192.4	1099.877	59746497.33	42094121.33	85985816	25247409.67	12017303.33	29097208.67
mango028314	MRF3	40.197	41.257	25.363	69.23	67.037	56.547	1408370.708	1592967.75	1269272.167	1986596.375	1940101.417	1813735.958
mango028849	Os02g0773300	13.667	18.103	22.68	7.217	9.337	7.893	1080241.396	933833.125	1019609.667	934037.188	718257.438	738553.396
mango029750	accB	27.31	38.997	52.19	19.623	19.493	22.76	1206027.833	1020100.625	1381977.667	771260.729	650144.271	757788.625
mango030037	ABCC8	18.903	2.487	0.28	1.193	4.807	4.217	363506.167	224265.036	86538.34	224485.807	159032.712	46309.887
mango031069	At1g06840	130.743	55.35	67.847	39.507	32.333	52.103	551076.917	466507.385	422713.052	458934.552	123791.948	245752.318
mango031169	RE	41.077	38.94	45.277	99.343	104.49	106.757	354674.052	398554.365	435910.01	418370.448	549467.938	559465.01
mango031532	ENO2	525.77	631.473	660.98	363.07	519.947	310.293	47112662.67	39513638.67	56960532	32152804.67	40807095.33	34221342
mango031829	VPS39	17.85	18.27	30.207	5.713	7.343	6.987	327551.771	415177.927	284328.375	407020.979	499444.552	324789.109
mango032009	SAC6	0.32	0.07	0.13	0.207	0.413	0.087	8726167.333	3168066.917	13242061	7535093	3510093.583	5190783
mango032095	exgA	2.463	2.453	12.647	1.543	5.523	4.377	726082.302	417935.958	605397.417	340726.146	316432.448	420246.802
mango032622	SPP2	106.24	100.547	169.077	28	61.943	60.087	3824173.833	3728226.667	4532774.75	2030338.542	1666033.333	1999753.458
mango033221	–	29.137	67.26	86.993	13.183	26.657	41.75	407787.885	164688.378	230557.328	216012.471	156628.076	102896.935
mango033409	FTSZ1	23.363	25.213	42.57	8.72	10.48	13.19	834630.917	637751.073	1093706.229	249376.729	228567.771	299652.328
mango034889	MTB	3.987	4.823	2.947	9.093	10.003	8.687	140038.404	125819.164	80701.871	142846.672	145785.974	115555.281
mango035105	SKP20	7.073	12.49	11.507	4.683	4.047	2.957	2534340.083	2353001.625	2375078.5	2237931.625	1963994.292	1739793.25
mango035149	At1g79260	14.97	26.47	26.68	4.42	10.743	11.28	2437932.083	2600219.792	1519332.604	2652060.667	1895509.958	2298095.917

**TABLE 3:** A total of 58 DEGs screened by WGCNA.

		DEGs express level					
Id	Symbol	RN0d	RN2d	RN6d	KT0d	KT2d	KT6d
mango000296	CYP98A2	9.077	45.163	7.223	28.54	135.043	29.203
mango000839	SAG113	932.533	670.88	624.033	254.743	335.347	309.333
mango003892	CYP75A1	3.343	75.497	13.91	35.847	129.567	34.317
mango004150	THFS	49.22	60	38.217	32.26	67.733	37.697
mango005596	CYP86A22	46.457	53.637	64.88	2.973	9.297	16.103
mango005775	ELF5	29.387	26.813	26.037	0.1	0.257	0.077
mango006296	4CL2	15.937	81.573	25.067	39.437	185.467	45.13
mango006430	GC5	28.737	35.537	45.947	16.92	19.077	14.26
mango006938	At3g47520	102.743	106.617	161.383	51.213	36.82	49.63
mango007487	UAH	9.41	12.19	7.76	5.117	10.513	5.913
mango008372	GLIP5	39.963	49.117	35.507	20.78	20.93	17.393
mango008492	CKL1	25.397	26.27	31.577	0.073	0.247	0.033
mango008818	BRIZ1	0.763	0.573	0.557	29.807	22.913	18.11
mango009044	–	68.547	109.253	32.673	43.683	132.607	40.69
mango011025	MC410	29.327	16.913	17.647	0.047	0.09	0
mango012295	RCOM_ 1506700	10.373	14.467	21.237	4.343	7.463	8.597
mango012298	MVD2	60.73	79.223	100.39	30.483	30.51	27.407
mango012341	SPBC776.07	4.817	5.737	3.673	9.85	9.967	9.53
mango012352	RTM2	0.507	0.387	3.96	0.253	0.073	0
mango012477	tal	393.79	559.74	637.897	233.253	289.607	195.803
mango013678	KAS1	52.033	76.32	97.343	39.407	26.873	28.02
mango013911	–	99.383	116.91	143.463	41.58	56.157	66.46
mango015130	SRK2A	13.223	8.907	10.103	39.007	35.01	35.897
mango017964	RTNLB8	17.583	23.377	14.03	9.523	25.83	13.333
mango018070	SUMO2	3.64	4.283	3.76	1.68	0.97	2.07
mango018604	GF14D	164.24	222.787	174.74	125.323	252.17	166.763
mango018984	PAE8	20.477	114.597	16.65	6.75	90.6	9.203
mango020228	AFB2	58.567	62.18	89.82	37.887	38.09	32.177
mango020536	DBR	14.047	3.567	1.4	2.497	4.22	5.127
mango021031	HEXO2	15.157	32.947	11.707	26.427	34.927	16.99
mango021699	PLT5	19.467	54.62	37.61	30.247	99.233	26.93
mango022055	ABCB1	22.193	17.357	14.127	31.92	29.63	28.647
mango022508	ADCK1	48.847	15.487	10.243	36.453	24.97	23.143
mango023351	HCS1	45.403	41.58	51.943	22.843	21.87	23.69
mango023529	XK2	11.023	14.383	24.583	8.207	15.717	13.29
mango023835	SCP2	65.787	92.243	79.777	19.347	51.837	41.58
mango024212	At5g42250	1.323	4.267	3.45	2.543	5.167	3.46
mango024861	TFT7	63.79	67.6	75.36	10.187	27.02	29.803
mango026117	PGL3	10.34	8.013	13.213	4.267	5.69	4.417
mango026233	POR1	171.973	175.867	175.513	71.66	87.68	90.32
mango027123	At2g47970	78.183	63.787	38.19	89.54	96.82	83.147
mango027688	EO	804.393	1005.29	2212.13	40.2	192.4	1099.877
mango028314	MRF3	40.197	41.257	25.363	69.23	67.037	56.547
mango028849	Os02g0773300	13.667	18.103	22.68	7.217	9.337	7.893
mango029750	accB	27.31	38.997	52.19	19.623	19.493	22.76
mango030037	ABCC8	18.903	2.487	0.28	1.193	4.807	4.217
mango031069	At1g06840	130.743	55.35	67.847	39.507	32.333	52.103
mango031169	RE	41.077	38.94	45.277	99.343	104.49	106.757
mango031532	ENO2	525.77	631.473	660.98	363.07	519.947	310.293
mango031829	VPS39	17.85	18.27	30.207	5.713	7.343	6.987
mango032009	SAC6	0.32	0.07	0.13	0.207	0.413	0.087
mango032095	exgA	2.463	2.453	12.647	1.543	5.523	4.377
mango032622	SPP2	106.24	100.547	169.077	28	61.943	60.087
mango033221	–	29.137	67.26	86.993	13.183	26.657	41.75
mango033409	FTSZ1	23.363	25.213	42.57	8.72	10.48	13.19
mango034889	MTB	3.987	4.823	2.947	9.093	10.003	8.687
mango035105	SKP20	7.073	12.49	11.507	4.683	4.047	2.957
mango035149	At1g79260	14.97	26.47	26.68	4.42	10.743	11.28

**TABLE 4:** A total of 31 DEPs screened by WGCNA.

Protein_id	Symbol	RN0d	RN2d	RN6d	KT0d	KT2d	KT6d
mango000296	CYP98A2	120449.669	336072.583	400800.042	254158.284	1019853.219	1696598.542
mango000839	SAG113	683416	124673.814	167780.609	434339.406	631877.458	455194.052
mango003892	CYP75A1	236681.745	1493866.958	2342342.417	339446.219	5058423.667	4947420.667
mango005596	CYP86A22	1515010.354	816778.292	253823.823	850163.25	169501.807	259646.812
mango006296	4CL2	267768.224	1657512.75	2795836.583	225517.089	6310093.667	7322742.833
mango006938	At3g47520	11182567.33	11850842	13976115.67	8119996.667	6680809.833	7291565.667
mango008372	GLIP5	639855.354	623948.417	819532.771	334714.906	301998.797	376429.344
mango008492	CKL1	425939.167	230623.167	228819.406	379393.182	145321.557	149100.297
mango009044	–	2286412.917	2726363.583	2348346.917	1447527.583	3331601.917	4125099.167
mango012295	RCOM_1506700	802806.979	708689.208	841368.208	675084.917	411557.135	555602.979
mango012298	MVD2	4598980.5	4295668.75	5038793	3759557.167	3429621.583	3287508.333
mango012477	tal	11053258	10469292	13088137	8039736	7254753.667	7475896.5
mango013678	KAS1	4089943.333	2669365.333	3076852	3255481.833	2750220.333	3036436.417
mango013911	–	3339037.083	3102724	2848404.833	3595689.167	1778566.875	2227503.875
mango020536	DBR	1327042.792	963911.958	489440.458	1111487.875	531932.365	427321.708
mango021031	HEXO2	151324.245	439039.583	416883.708	372989.229	879524.708	819515.25
mango021699	PLT5	667121.625	738229.729	662353.208	269335.333	1002994.146	1141336.271
mango022508	ADCK1	147165.857	192104.661	181896.562	156913.062	298447.896	278558.891
mango023351	HCS1	520459.594	426767.615	297574.5	490239.083	337919.406	299679.25
mango023529	XK2	4484826.25	3297040.083	5104332.333	3658099.167	3117518.25	3054485.167
mango023835	SCP2	1972901.833	1787336.958	1749565.875	2421409.417	1098470.312	1508311.292
mango024212	At5g42250	197642.305	342618.052	528385.219	160343.128	154011.594	295552.198
mango024861	TFT7	730657.021	629114.354	746246.938	404433.219	380753.344	407370.042
mango026233	POR1	5275425.5	5293905.667	6443040.667	3263134.917	3106708.333	3300088.25
mango027123	At2g47970	984970.667	688723.688	593986.25	568024.156	611011.042	705327.167
mango027688	EO	59746497.33	42094121.33	85985816	25247409.67	12017303.33	29097208.67
mango029750	accB	1206027.833	1020100.625	1381977.667	771260.729	650144.271	757788.625
mango030037	ABCC8	363506.167	224265.036	86538.34	224485.807	159032.712	46309.887
mango031069	At1g06840	551076.917	466507.385	422713.052	458934.552	123791.948	245752.318
mango032622	SPP2	3824173.833	3728226.667	4532774.75	2030338.542	1666033.333	1999753.458
mango033409	FTSZ1	834630.917	637751.073	1093706.229	249376.729	228567.771	299652.328

Among the DEGs obtained in WGCNA, three are related to plant hormone signal transduction (*SAG113*, *SRK2A*, and *ABCB1*). One gene, which was annotated as GSH dehydrogenase (*At5g42250*), is related to the regulation of redox homeostasis in plant cells and involved in GSH metabolism. One protein (*SAG113*) related to plant hormone signal transduction and one (*At5g42250*) regulating cell redox homeostasis were also found in the 31 DEPs obtained in WGCNA. Notably, two DEPs, namely, *SAG113* and *At5g42250*, ran through the transcriptome and proteome of the study and were annotated to GSH metabolism and plant hormone signal transduction. Thus, these pathways may be important means for mango resistance to *Xcm*.

### Real-time polymerase chain reaction validation

We performed real-time polymerase chain reaction analysis of three plant hormone-related DEGs/DEPs obtained by WGCNA to validate the RNA-seq results, and these genes showed different expression patterns at 0, 2, and 6d. The expression patterns of these genes obtained by qRT-PCR largely confirmed the transcriptome data (Supplementary Figure 4).

## Discussion

In our study, RNA-seq and DIA techniques were used to holographically identify the changes in mRNA and protein levels in mango at different stages of *Xcm* infection. A total of 28,704 RNA data and 12,877 protein information were obtained. After differential analysis, 14,397 DEGs and 3,438 DEPs were obtained, and a large number of differential genes were shared between the two varieties of mango. This paper focused on DEGs/DEPs involved in redox homeostasis regulation and plant hormone signal transduction in mango cells. Given the great contribution of SOD and CAT in plant oxidative stress, we analyzed the correlation between the changes in SOD and CAT levels and changes in transcriptome and proteome in mango to explore the important genes related to trait indicators.

Photosynthesis, GSH metabolism, and peroxisomes play important regulatory roles in plant cell redox homeostasis during plant resistance to pathogens. Photosynthesis is an important source of ATP and carbohydrates in plants. A series of genes involved in photosynthesis can participate in the production and signal transduction of plant hormone signaling molecules, such as ABA, ETH, IAA, GA, etc., and the production and signal transduction of non-hormone signaling molecules. PSI and PSII are the main sources of ROS production and play a crucial role in the balanced synthesis of ROS and NO (Apel and Hirt, 2004; Asada, 2006; Del Río, 2015; Lu and Yao, 2018). In mango, more than 80% of DEGs and DEPs involved in PSI and PSII were generally up-regulated in KT and RN. However, the change in RN was always negligible, and that of related genes in KT continuously increased (Table 5). The general imbalance of photosynthetic genes may hinder the stability of photosynthesis in susceptible plants; it was also encountered in chickpeas infected with *F. oxysporum f.* sp. *ciceri* race 1 (Bhar et al., 2017) and *Cucurbita ficifolia* Bouché infected with *Fusarium oxysporum f*. sp. *cucumerinum*. In addition, DEGs *FD3* and *Os07g0147900*, which were annotated as Fd, were up-regulated in RN and down-regulated in KT, and DEPs, including *FD3*, *SIR1*, and *FTRC*, were annotated as Fd and up-regulated in RN and KT. However, the change in RN was more significant.

**TABLE 5:** DEGs and DEPs in photosystem 1 and photosystem 2.

		DEGs express level					
Id	Symbol	RN0d	RN2d	RN6d	KT0d	KT2d	KT6d
mango000380	PSAO	28.067	46.73	42.25	5.197	100.017	121.05
mango003053	psaD	0.063	0.04	0.103	0.093	2.73	2.85
mango006427	PSAH2	3.143	4.42	1.913	6.647	35.077	30.883
mango007574	psaD	84.55	102.213	125.847	32.657	96.63	155.057
mango010109	PSAN	4.937	15.983	7.387	2.78	44.307	73.97
mango015793	PSBY	0.793	1.65	0.93	0.66	18.933	39.063
mango018113	PSAE	20.483	19.163	14.077	12.933	35.727	52.393
mango018135	PSB28	15.29	31.427	34.053	2.483	12.833	10.91
mango019341	PSBW	11.713	17.093	14.37	0.83	16.127	18.343
mango019347	PSBW	0	0	0.203	0.97	23.453	39.36
mango020695	PSBW	133.573	198.41	336.833	61.183	226.493	252.927
mango020754	PSAL	43.027	55.49	36.457	17.483	149.747	210.073
mango025005	PSBS	50.043	72.147	99.383	18.66	75.52	61.463
mango026466	PSBY	32.703	50.273	48.007	10.027	58.307	78.193
mango026761	PSB28	1.143	3.85	2.467	0.677	5.683	2.937
mango026786	PSAEA	66.377	78.883	71.193	23.287	79.123	117.487
mango029879	PSAH2	12.33	26.457	20.117	5.76	58.56	66.347
mango030680	PSAF	95.027	140.253	162.19	42.743	134.8	146.973
mango032227	PSAN	0	0.053	0	0	0.2	0.877
		**DEPs express level**					
**Protein_id**	**Symbol**	**RN0d**	**RN2d**	**RN6d**	**KT0d**	**KT2d**	**KT6d**
mango006427	PSAH2	2701098.292	712847.542	1326046.208	13895611.17	4650683.375	10251425.67
mango029879	PSAH2	2701098.292	712847.542	1326046.208	13895611.17	4650683.375	10251425.67
mango007574	psaD	6829527.667	3117529.667	3623329.292	31275804.67	14579772.67	21904209.33
mango010025	psaA	11205578.5	3604213.917	3367035.833	44034390.67	20162870	35687958.67
mango015142	LHCA3	2531327.75	668129.188	918001.333	13884698.33	5543263.167	8379549.833
mango015191	LHCA3	2531327.75	668129.188	918001.333	13884698.33	5543263.167	8379549.833
mango016666	psbB	7404652.833	4324037.917	4967177.083	15096296	10150018.17	15987511.67
mango018113	PSAE	266476.005	205574.255	56204.501	2521959.5	793993.719	1646594.167
mango020436	PSB27-1	2957401.5	1727613.333	1635928.833	3728865.583	2361803.708	2865011.208
mango020754	PSAL	219881.568	82765.891	108931.694	765285.792	424844.661	754995.906
mango022823	psbB	3362773.917	1869271.75	1268698.5	6328656.917	3476546.333	5686411.5
mango023837	LHCA5	35759.461	NA	1.032	190346.31	621114.688	262428.484
mango026786	PSAEA	1427598.792	788556.125	475287.797	6385375.833	2773814.167	5008947.25
mango030680	PSAF	13723842.33	4954353.583	6383658.583	50647948	21665666	38667322.67
mango032227	PSAN	6035151.833	1928648.958	2417696.083	25466472	8873263.667	12152935.67

The plant-type redox system composed of Fd-NADP (+) reductase and its redox partner Fd can play an important role in plant-pathogen interaction (Iyanagi, 2022). Fd can interact with the HC-Pro protein of *sugar cane mosaic virus* (*SCMV*) in maize infected with *SCMV* and may interfere with the post-translational modification of Fd in the chloroplast of maize sheath cells, which will disturb chloroplast structure and function (Cheng et al., 2008). Fd may activate hypersensitivity related events, such as H_2_O_2_ accumulation, through the recognition of interacting proteins in mango to enhance the plant’s resistance. Overexpression of Fd also enhances the resistance of *Arabidopsis* (Ger et al., 2014), sweet pepper (Dayakar et al., 2003), and tobacco (Huang et al., 2007).

Glutathione metabolism is the metabolic process of gamma-glutamyl-cysteinyl-glycine (GSH) in plants. GSH is an antioxidant that can resist free radical damage, support the dynamic relationship with ROS, redox regulation, and signal transduction, and protect cells from external factors (Diaz-Vivancos et al., 2015; Noctor et al., 2023). GSH activates the potato defense system by reducing potential damage to host cells in *Potato virus* Y NTN medical record system, which results in reduced virus concentration and limits systemic infection of potatoes caused by oxidative stress (Otulak-Kozieł et al., 2022). In our study, 81 genes were involved in GSH metabolism, of which 20 genes, such as L-ascorbate peroxidase (*APX1*), were consistently expressed at mRNA and protein levels. GST (*HSP26-A*, *PARC*, *GSTU8*, and *GSTL3*) and GSH dehydrogenase/transferase (*DHAR2*) were down-regulated in resistant and susceptible mango cultivars. Glucose-6-phosphate 1-dehydrogenase (*G6PD2* and *G6PD4*) and GPX (*GPX2*) were up-regulated, and the expression trends of the three genes in the two omics were opposite. Gamma-glutamyltranspeptidase (*GGT2*) was down-regulated in the transcriptome and up-regulated at the protein level. Two GSTs (*GSTF11* and *GSTU17*) showed the opposite result, and one GSH dehydrogenase (*At5g42250*) was found in WGCNA and differentially expressed in the two omics. Thus, the metabolic process of GSH is very important for mango to resist *Xcm* invasion, and its related genes regulate its metabolic process at different levels, thus protecting mango cells from the oxidative stress caused by *Xcm* infection.

Peroxisome is an important organelle in ROS metabolism, mainly producing superoxide anion (O_2_^–^) and hydrogen peroxide (H_2_O_2_) (Corpas et al., 2020). Peroxisome is involved in a series of ROS generation and scavenging mechanisms, and participates in programmed cell death of plant cells to resist environmental stresses (Huang et al., 2022). In our study, at the transcriptome level, nearly half of the peroxisome-related DEGs changed more significantly in RN, whereas at the proteome level, 30 out of 38 DEPs, including POD (*CAT1*) and SOD (*FSD2*), were up-regulated in RN, and the change range was more than that observed KT. Thus, several multifunctional genes can regulate the balance of ROS in mango by positively regulating the biosynthesis of peroxisomes.

Hormones play a vital role in plant disease resistance. The signaling pathways of multiple hormones are not independent of each other in the disease resistance response; however, the interaction between hormones forms a complex regulatory network that enables plants to efficiently coordinate different hormones in the body to improve plant resistance, which is an effective method to resist pathogen invasion (Verma et al., 2016). In this study, a large number of genes were found to be involved in the signal transduction of plant hormones, such as IAA, ABA, GA, ETH, and so on, at the transcriptional and protein levels. The expression levels of these plant hormone-related DEGs in RN were relatively low and generally down-regulated. The related proteins with large differences in expression in the proteome were similar to the transcriptome, mainly that of KT. Through cross-validation of transcriptome and proteomics and WGCNA, three key genes (*SAG113*, *SRK2A*, and *ABCB1*) that were co-expressed in two groups were finally screened out, and they were related to the changes in SOD and CAT activities. *SAG113* was also identified in WGCNA of the proteome. Thus, these three genes are not only involved in the signal transduction of plant hormones but may also regulate the redox homeostasis of mango cells during stress response through signal transduction.

## Conclusion

The response of mango fruit to Xcm is a complex process, and our understanding of MBLS pathology is limited. The symptoms of disease-resistant varieties and susceptible varieties appeared on the 2nd day and differentiated on the 6th day of the experiment. To determine the positively or negatively affected genes, we mainly analyzed the significantly DEGs that maintained a consistent trend from day 0 to day 6. The genes and proteins identified in this study provide valuable resources for mango resistance to MBLS breeding and can benefit researchers in this field.

## Data availability statement

The authors acknowledge that the data presented in this study must be deposited and made publicly available in an acceptable repository, prior to publication. Frontiers cannot accept a manuscript that does not adhere to our open data policies.

## Author contributions

FL and R-LZ contributed to the conception and design of the study. XS wrote the first draft of the manuscript. QY and KZ provided scientific advice. LW performed the statistical analysis. All authors contributed to the manuscript revision and read and approved the submitted version.

## Footnotes

1
https://ngdc.cncb.ac.cn/search/?dbld=gwh&q=PRJCA002248

2
http://www.geneontology.org/

3
https://www.kegg.jp

## Supplementary material

Supplementary Figure 1 Venn diagram of transcriptome sample abundance. Each set in the figure is filled with different colors and labeled with the number of genes contained in each set. The overlapping parts of the set represent the number of genes shared between groups, while the non-overlapping parts represent the unique genes of each group.

Supplementary Figure 2 Venn diagram of proteome sample abundance. Each group in the figure is filled with a different color and marked with the number of proteins each group contains. The overlapping part of the set represents the number of proteins shared between groups, and the non-overlapping part represents the genes unique to each group.

Supplementary Figure 3 Changes of SOD and CAT activity in fruits of different mango varieties under Xcm stress. The mark above the error line is the difference analysis result between the treatment group and the control group.

Supplementary Figure 4 Validation of the transcriptome data. The ordinate in the figure represents the relative expression of differentially expressed genes in qRT-PCR. The abscissa represents the different treatment groups; Error bars are standard deviations.

Supplementary Table 1 Primer sequences used for qRT-PCR.

Supplementary Table 2 GO annotation results of DEGs.

Supplementary Table 3 Results of KEGG enrichment analysis of DEGs.

Supplementary Table 4 Genes involved in plant hormone signaling.

Supplementary Table 5 Redox-related genes.

Supplementary Table 6 GO annotation results of DEPs.

Supplementary Table 7 Results of KEGG enrichment analysis of DEPs.

Supplementary Table 8 Proteins involved in plant hormone signaling.

Supplementary Table 9 Redox-related proteins.

Supplementary Table 10 Cross-validation of transcriptomics and proteomics.
