# Supplementary figures and images for: Transcriptomic and proteomic analyses of Mangifera indica in response to Xanthomonas critis pv. mangiferaeindicae
Source: Front Microbiol. 2023 Jul 4;14:1220101. doi: 10.3389/fmicb.2023.1220101 (PMC10352610; doi:10.3389/fmicb.2023.1220101)

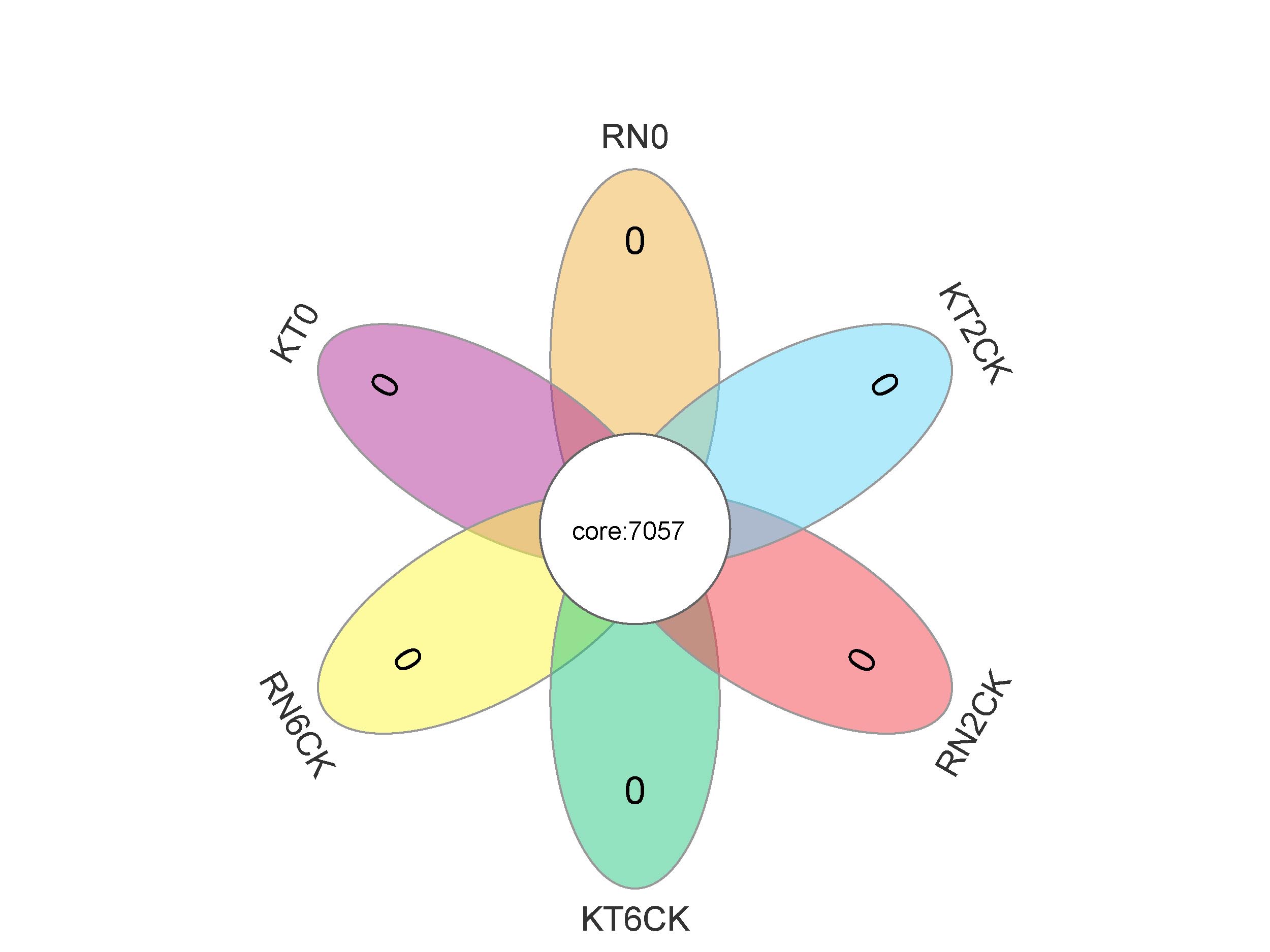

Supplement: Supplementary Figure 1 — Venn diagram of transcriptome sample abundance. Each set in the figure is filled with different colors and labeled with the number of genes contained in each set. The overlapping parts of the set represent the number of genes shared between groups, while the non-overlapping parts represent the unique genes of each group. [file Image_1.JPEG]

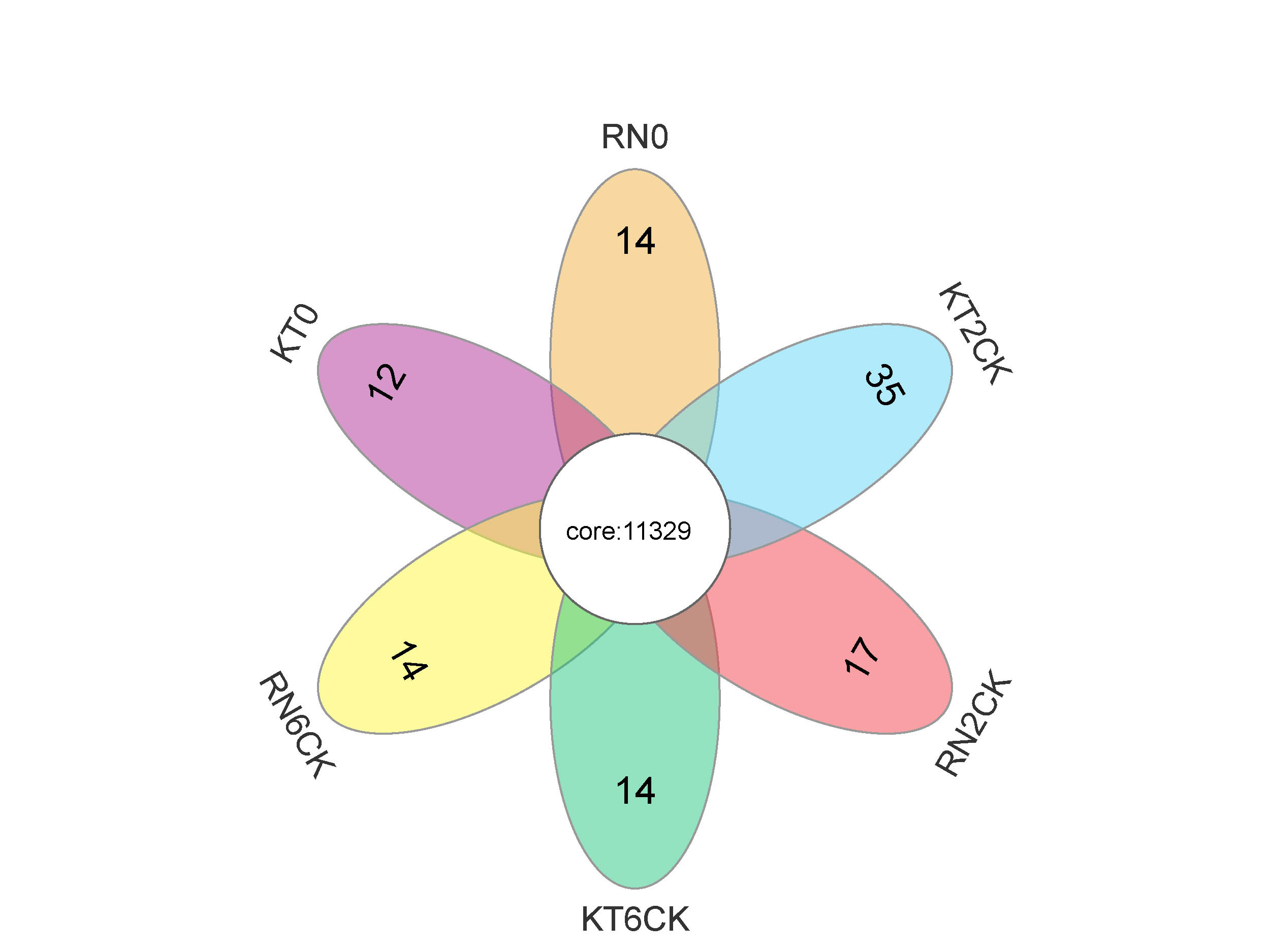

Supplement: Supplementary Figure 2 — Venn diagram of proteome sample abundance. Each group in the figure is filled with a different color and marked with the number of proteins each group contains. The overlapping part of the set represents the number of proteins shared between groups, and the non-overlapping part represents the genes unique to each group. [file Image_2.JPEG]

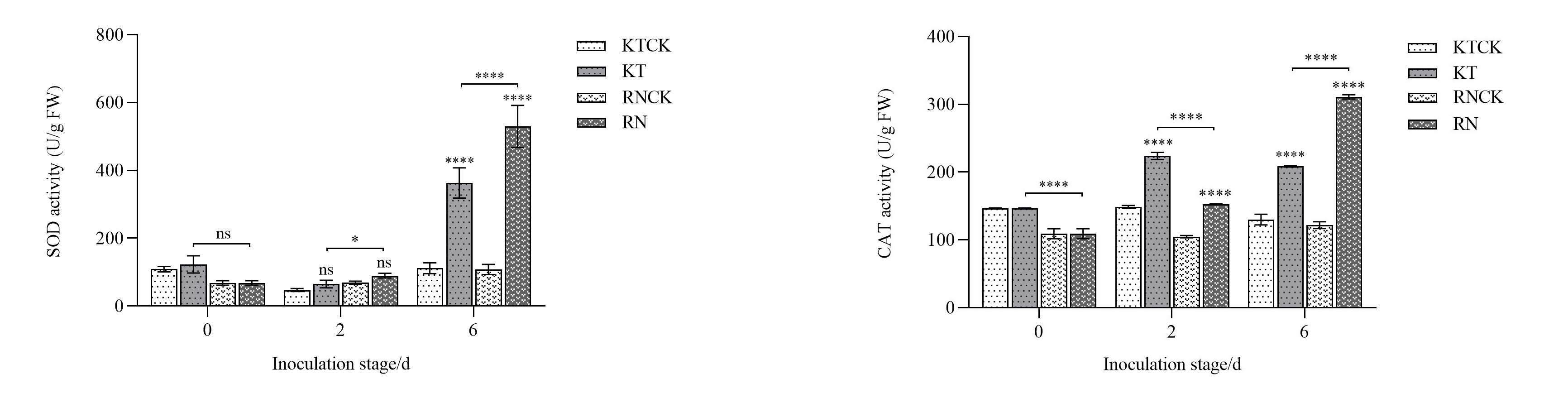

Supplement: Supplementary Figure 3 — Changes of SOD and CAT activity in fruits of different mango varieties under Xcm stress. The mark above the error line is the difference analysis result between the treatment group and the control group. [file Image_3.JPEG]

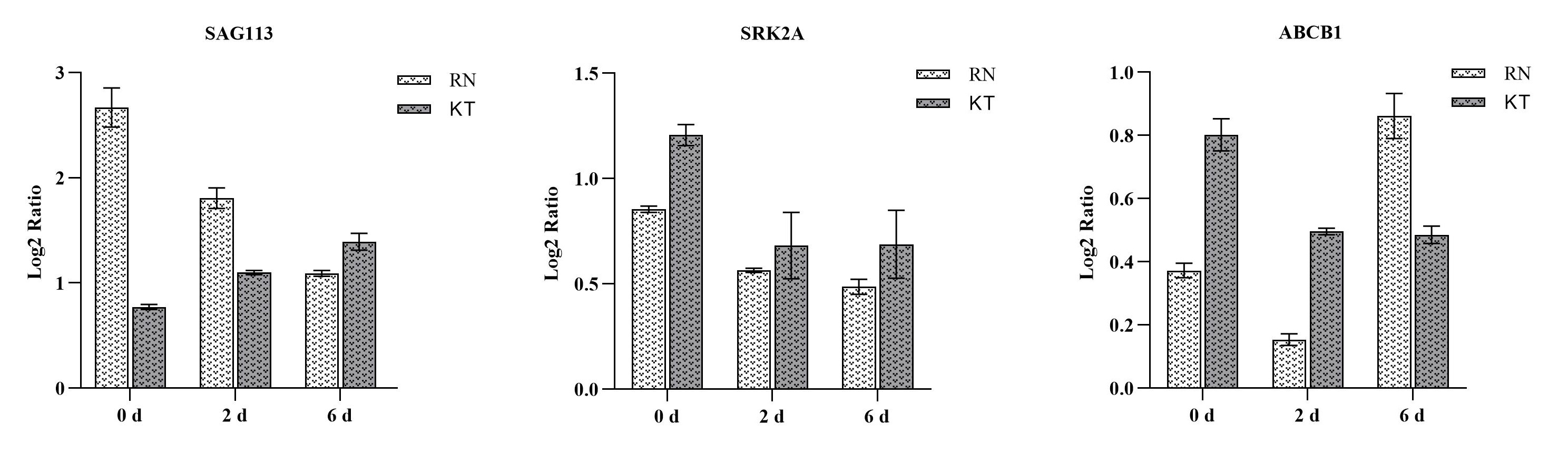

Supplement: Supplementary Figure 4 — Validation of the transcriptome data. The ordinate in the figure represents the relative expression of differentially expressed genes in qRT-PCR. The abscissa represents the different treatment groups; Error bars are standard deviations. [file Image_4.JPEG]
